# Prenatal Exposure to Phthalates and Infant Development at 6 Months: Prospective Mothers and Children’s Environmental Health (MOCEH) Study

**DOI:** 10.1289/ehp.1003178

**Published:** 2011-10-01

**Authors:** Yeni Kim, Eun–Hee Ha, Eui–Jung Kim, Hyesook Park, Mina Ha, Ja–Hyeong Kim, Yun–Chul Hong, Namsoo Chang, Bung–Nyun Kim

**Affiliations:** 1 Department of Adolescent Psychiatry, National Center for Child and Adolescent Psychiatry, Seoul National Hospital, Seoul, Korea; 2 Department of Preventive Medicine, Ewha Womans University, College of Medicine, Seoul, Korea; 3 Department of Psychiatry, Ewha Womans University, College of Medicine, Seoul, Korea; 4 Department of Preventive Medicine, Dankook University, College of Medicine, Cheonan, Korea; 5 Department of Pediatrics, University of Ulsan, College of Medicine, Ulsan, Korea; 6 Department of Preventive Medicine, Seoul National University, College of Medicine, Seoul, Korea; 7 Department of Nutritional Science and Food Management, Ewha Womans University, College of Health Sciences, Seoul, Korea; 8 Division of Child and Adolescent Psychiatry, Department of Psychiatry, Seoul National University, College of Medicine, Seoul, Korea

**Keywords:** development, dibutyl phthalate, di(2-ethylhexyl) phthalate, infant, prenatal

## Abstract

Background: There are increasing concerns over adverse effects of prenatal phthalate exposure on the neurodevelopment of infants.

Objectives: Our goal was to explore the association between prenatal di(2-ethylhexyl) phthalate and dibutyl phthalate exposure and the Mental and Psychomotor Developmental Indices (MDI and PDI, respectively) of the Bayley Scales of Infant Development at 6 months, as part of the Mothers and Children’s Environmental Health Study.

Methods: Between 2006 and 2009, 460 mother–infant pairs from Seoul, Cheonan, and Ulsan, Korea, participated. Prenatal mono(2-ethyl-5-hydroxyhexyl) phthalate (MEHHP), mono(2-ethyl-5-oxohexyl) phthalate (MEOHP), and mono-*n*-butyl phthalate (MBP) were measured in one urine sample acquired from each mother during the third trimester of pregnancy. Associations with log-transformed creatinine-corrected phthalate concentrations were estimated using linear regression models adjusted for potential confounders.

Results: MDI was inversely associated with the natural log concentrations (micrograms per gram creatinine) of MEHHP [β = –0.97; confidence interval (CI), –1.85 to –0.08] and MEOHP (β = –0.95; CI, –1.87 to –0.03), and PDI was inversely associated with MEHHP (β = –1.20; CI, –2.33 to –0.08). In males, MDI was inversely associated with MEHHP (β = –1.46; CI, –2.70 to –0.22), MEOHP (β = –1.57; CI, –2.87 to –0.28), and MBP (β = –0.93; CI, –1.82 to –0.05); PDI was inversely associated with MEHHP (β = –2.36; CI, –3.94 to –0.79), MEOHP (β = –2.05; CI, –3.71 to –0.39), and MBP (β = –1.25; CI, –2.40 to –0.11). No significant linear associations were observed for females.

Conclusions: The results suggest that prenatal exposure to phthalates may be inversely associated with the MDI and PDI of infants, particularly males, at 6 months.

Recent evidence suggests that environmental pollutants can be detrimental to the neurocognitive development of children (Bellinger 2008). Such evidence is especially strong in the case of lead, which has been shown to manifest detrimental effects on intelligence (Canfield et al. 2003) and increase hyperactivity and impulsivity in children (Braun et al. 2006; Kim et al. 2010). The exposure of children to phthalates has raised concerns, because these chemicals have been associated with developmental and reproductive toxic effects in laboratory animals (Borch et al. 2006; Gray et al. 2000; Ishido et al. 2004b). Previous animal studies have reported that phthalates cause hyperactivity and impulsivity in rats; which appears similar to the clinical features of attention deficit hyperactivity disorder (ADHD), a condition most commonly identified in school-aged children (Ishido et al. 2004b; Masuo et al. 2004). A recent cross-sectional survey reported associations between phthalate metabolites and intelligence scores (Cho et al. 2010) and ADHD symptoms in school-aged children (Kim BN et al. 2009).

Phthalates—diesters of 1,2-benzenedicarboxylic acid (phthalic acid)—are a group of synthetic chemicals with a wide spectrum of industrial and commercial uses, including primary plasticizers for polyvinyl chloride and solvents in personal care products (Wormuth et al. 2006). Phthalate plasticizers are slowly emitted into the surrounding environment (Wormuth et al. 2006), constituting an indoor pollutant (Bornehag et al. 2005). Phthalates can be ingested through food or inhaled through contaminated air or dust. Dermal contact with care products that contain phthalates and medical devices contaminated with phthalates are another possible source of exposure (Hernandez-Diaz et al. 2009). After entering the body, phthalates undergo rapid metabolism to monoesters and can also be oxidized further to oxidative metabolites (Engel et al. 2010).

Phthalates are suspected to interfere with the thyroid hormone system (Ghisari and Bonefeld-Jorgensen 2009; Huang et al. 2007), a system vital to normal brain development in the fetus and infant (Berbel et al. 2010). The maternal transmission of phthalates to offspring has been demonstrated; these compounds have been found in the amniotic fluid and fetal circulation in humans (Huang et al. 2009; Wittassek et al. 2009). It has been estimated that infants may experience higher exposures to phthalates in relation to their body weight (Wormuth et al. 2006). All of these findings suggest that phthalates may cause disturbances in the normal developmental trajectory of the fetal and infant brain (Tanida et al. 2009). Prenatal exposure to phthalates has been associated with poor birth outcomes (Wolff et al. 2008), neurological outcomes in the neonate (Engel et al. 2009), behavioral problems (Engel et al. 2010), reduced masculine play in boys (Swan et al. 2010), and social impairment (Miodovnik et al. 2011) in childhood. However, the effects of prenatal phthalate exposure on neurodevelopment at infants at 6 months have not been investigated.

In this study, we hypothesized that prenatal exposure to di(2-ethylhexyl) phthalate (DEHP) and dibutyl phthalate (DBP) would be inversely associated with Mental and Psychomotor Developmental Indices (MDI and PDI, respectively), as measured by the Korean Bayley Scales of Infant Development, 2nd edition (BSID-II) at 6 months (Bayley 1993; Park and Cho 2006).

## Methods

*Recruitment of participants.* This study was part of the Mothers and Children’s Environmental Health Study (MOCEH), an ongoing multicenter prospective cohort study of environmental factors contributing to the health of mothers and children. The study protocols, which were approved by the Institutional Review Boards of Ewha Womans University, Dankook University Hospital, and Ulsan University Hospital, are described in detail elsewhere (Kim BM et al. 2009). In brief, pregnant women in their first trimester were approached for recruitment at obstetric clinics located in Seoul (a metropolitan city with population of 10,464,051 and a population density of 16,586/km^2^), Cheonan (a mixture of rural and high-tech industrial area with population of 570,107 and a population density of 895/km^2^), and Ulsan (an industrial area with population of 1,129,962 and a population density of 1,034/km^2^). The inclusion criteria were pregnant women who were age > 18 years and residence at the targeted study site at the time of enrollment. Eligible pregnant women visiting the obstetric clinics were invited to participate through posters on the walls of the clinic and by the examining obstetric doctors. All study participants provided written informed consent at enrollment. From 2006 through 2009, 718 children (50.0% of enrolled children were followed at 6 months) were examined at 6 months of age.

*MDI and PDI assessed at 6 months.* The BSID-II produces indicators of infant neurodevelopment from 0 to 3 years of age (Bayley 1993). Each test produces developmental indices (composite scores that compare developmental performance of a child with the norms taken from typically developing Korean children of the same age), which are expressed as the MDI and the PDI (Park and Cho 2006). The BSID-II was conducted in a quiet room by trained examiners for 30 to 45 min. Training on the BSID-II was coordinated by a specialist (interrater reliability: kappa value > 0.8) before the beginning of the evaluation of the infants. The intelligence of the mother was measured using the short form of the Korean Wechsler Adult Intelligence Scale (Um et al. 1992). Scores obtained from this abbreviated version of the test show very good correlation with the Full-Scale Wechsler Adult Intelligence Scale score (Lim et al. 2000; Silverstein 1990). There were three to five raters trained to perform the assessment at each center (Seoul, Cheonan, and Ulsan). Interrater reliability was confirmed annually through rater training sessions and video monitoring of the examination process. All test procedures and interpretation of the results were conducted according to The Standards for Educational and Psychological Testing (American Educational Research Association et al. 1999).

*Prenatal phthalate exposure during pregnancy.* Prenatal phthalate exposure was determined by measuring phthalate metabolites in the spot urine of the mother during the third trimester of pregnancy (range of gestational age at urine collection: 35.7–41.7 weeks). The spot urine samples were collected at the obstetric clinics between 0900 and 1800 hours. We measured the secondary metabolites of DEHP [mono(2-ethyl-5-hydroxyhexyl) phthalate (MEHHP); mono(2-ethyl-5-oxohexyl) phthalate (MEOHP)] and DBP [mono-*n*-butyl phthalate (MBP)]. Samples were refrigerated at –20°C. The measurement process used to quantify phthalate metabolites has been described in detail in the Centers for Disease Control and Prevention (CDC) Laboratory Procedure Manual (CDC 2009). The monoester phthalates were measured using high-performance liquid chromatography tandem mass spectrometry (Agilent 6410 Triple Quad LCMS; Agilent, Santa Clara, CA, USA). One reagent blank and one quality control sample were analyzed simultaneously with each batch of samples. The quality control samples were spiked with pooled urine and a mixture of phthalate monoester standards (100 ng/mL). The between-day coefficient of variation for the assay ranged from 0.5% to 8.9%. Creatinine concentration was measured using an enzymatic reaction with CREA reagent (Roche, Basel, Switzerland) on a Hitachi 7600 II analyzer (Hitachi, Tokyo, Japan). The limits of detection (LODs) of the phthalate metabolites were 0.056, 0.049, and 0.440 µg/L for MEHHP, MEOHP, and MBP, respectively. The LOD was defined as the concentration of phthalates that could be detected with a signal-to-noise ratio of 3 (Boque and Heyden 2009). Concentrations of phthalate metabolites below the LOD were imputed with a value equal to LOD/2 (Hornung and Reed 1990).

*Statistical analysis.* All statistical analyses were performed using SPSS 15.0 for Windows (SPSS, Chicago, IL, USA). Characteristics of the study subjects with respect to maternal phthalate exposure status were analyzed using chi-square tests or *t*-tests. The associations between maternal urine phthalate concentration [MEHHP, MEOHP, the molar sum of MEHHP and MEOHP (HHP + OHP, micromoles per liter), and MBP] and the MDI and PDI were examined using a linear regression model (α = 0.05). To improve the linearity of the modeled relationship, we used natural log-transformed values for the analysis of phthalate metabolites (Wolff et al. 2008). Creatinine-corrected concentrations of urinary phthalate metabolites (micrograms per gram creatinine) were used to normalize for urine dilution (Cho et al. 2010; Wolff et al. 2008; Ye et al. 2008). The model that used natural log-phthalate biomarkers with the natural log-creatinine in the model produced similar results (data not shown). To select covariates for inclusion in the multivariate models, we searched the literature to identify risk factors associated with phthalate exposure or infant neurodevelopment. The key covariates used in this study were the following: infant sex, birth weight, maternal age at delivery, maternal education level, family income, breast-feeding status, and residential area (Cho et al. 2010; Kim BN et al. 2009; Kim Y et al. 2009). The covariates were based on self-reported information given during the study interview. The variables were categorical and modeled using categories shown in Table 1. We also examined the interaction between infant sex and phthalate (α = 0.10). For the mothers whose intelligence was measured, a subgroup analysis was performed to further adjust for maternal intelligence. For the categorical analysis, quartiles of the phthalate biomarkers were created using the creatinine-corrected value.

**Table 1:** Demographic characteristics of the sample.

Residential area		
Characteristic	Total (*n *= 460)	Cheonan (*n *= 113)	Seoul (*n *= 95)	Ulsan (*n *= 252)	*p-*Value
No. of female infants (%)	225 (48.9)		64 (56.6)	48 (51.1)	113 (45.0)		0.11
MDI	96.7 ± 12.0		96.1 ± 10.7	92.3 ± 15.6	98.7 ± 12.6		0.01
PDI	96.5 ± 15.2		97.7 ± 14.5	88.9 ± 17.5	98.8 ± 13.7		< 0.01
Birth weight [g (mean ± SD, *n*)]	3273 ± 377, 455		3266 ± 320, 113	3280 ± 387, 90	3273 ± 397, 252		0.96
Maternal age at delivery [years (mean ± SD, *n*)]	29.9 ± 3.4, 457		29.1 ± 3.5, 113	31.5 ± 3.4, 93	29.7 ± 3.2, 251		< 0.01
Breast-feeding [*n* (%)]							0.09
< 1 month	165 (37.2)		32 (29.1)	31 (36.5)	102 (41.1)		
> 1 month	278 (62.8)		78 (70.9)	54 (63.5)	146 (58.9)		
Not specified	17 (3.7)		3 (2.7)	10 (10.5)	4 (1.9)		
Yearly household income [*n* (%)]	< 0.01						
< $2,000	42 (9.1)		12 (10.6)	1 (1.1)	29 (11.5)		
$2,000–$4,000	334 (72.6)		77 (68.1)	64 (67.4)	193 (76.6)		
≥ $4,000	71 (15.4)		17 (15.0)	28 (29.5)	26 (10.3)		
Not specified	13 (2.8)		7 (6.2)	2 (2.1)	4 (1.6)		
Maternal education [*n* (%)]							< 0.01
≤ High school	132 (28.7)		43 (38.1)	13 (13.7)	76 (30.2)		
College	76 (16.5)		16 (14.2)	11 (11.6)	49 (19.4)		
≥ University	229 (49.8)		50 (44.2)	60 (63.2)	119 (47.2)		
Not specified	23 (5.0)		4 (3.5)	11 (11.6)	8 (3.2)		
MEHHP [µg/L (mean)]*a,b,c*	8.9		11.0	10.8	7.5		0.02
25th percentile	4.3		6.1	5.6	3.6		
50th percentile	10.1		11.6	14.3	7.8		
75th percentile	21.4		22.1	28.3	18.2		
MEOHP [µg/L (mean)]*a,b,c*	7.4		10.4	8.7	6.0		0.01
25th percentile	3.8		5.0	4.4	2.7		
50th percentile	7.9		10.0	10.5	6.4		
75th percentile	17.1		18.9	24.5	14.8		
MBP [µg/L (mean)]*a,b,c*	12.4		15.3	10.4	12.1		0.04
25th percentile	5.4		10.2	3.5	4.3		
50th percentile	16.6		15.4	15.1	18.7		
75th percentile	41.1		23.6	44.6	47.2		
Creatinine [mg/L (mean)]*a*	62.8		64.2	61.4	62.8		0.64
25th percentile	38.9		42.2	32.4	41.2		
50th percentile	69.0		70.3	67.1	69.0		
75th percentile	111		109	121	104		
Gestational age at urine collection [weeks (mean)]*a*	39.4		39.5	39.4	39.4		0.71
25th percentile	38.6		36.6	38.6	38.7		
50th percentile	39.4		39.4	39.3	39.4		
75th percentile	40.3		40.4	40.1	40.3		
**a**Presented as geometric mean. **b**Number of samples below LOD was 27 (5.9%) for MEHHP, 27 (5.9%) for MEOHP, and 43 (9.3%) for MBP metabolites. **c**Geometric SD was 3.9 (range, 0.28–303.6 µg/L) for MEHHP, 3.7 (range, 0.25–262.8 µg/L) for MEOHP, and 5.7 (range, 0.22–528.1 µg/L) for MBP.							

## Results

*Participant characteristics.* The geographic distribution of MOCEH study participants whose infants were examined at 6 months of age with BSID-II was as follows: Cheonan, *n* = 178 (24.8%); Seoul, *n* = 203 (28.3%); and Ulsan, *n* = 337 (46.9%). From the 718 subjects, we excluded preterm deliveries (birth before 35 weeks, *n* = 12) and twin births (*n* = 15) (Kim Y et al. 2009). Of the resulting 691 mothers, 460 (66.6%) had provided sufficient urine samples during the third trimester of pregnancy for the analysis of phthalates. Therefore, the data of 460 mother–infant pairs were included in the final analysis. No significant differences were found in the background characteristics between the children included (*n* = 460) and excluded (*n* = 231) from the analysis because of lack of urine phthalate exposure information (data not shown), except for maternal age. The mothers of the children included in the analysis (29.9 ± 3.4 years, *n* = 457) were younger than the mothers of the children not included in the analysis (30.8 ± 3.9 years, *n* = 227; *p* = 0.002).

The mean MDI for the total sample (*n* = 460) was 96.7 (range, 50–133), and the mean PDI was 96.5 (range, 50–134). The mean (± SD) MDI was 96.3 ± 12.2 for male infants and 97.1 ± 11.9 for female infants, and the mean PDI was 94.8 ± 15.0 for male infants and 98.2 ± 15.3 for female infants. The female infants had significantly higher PDI scores than did the male infants (*p* = 0.02). The PDI scores of the male and female infants at the Ulsan center were significantly different (males = 96.2, females = 101.9, *p* < 0.01); however, there was no difference between PDI scores of male and female infants at the centers in Cheonan (males = 96.2, females = 97.7, *p* = 0.9) and Seoul (males = 87.5, females = 90.3, *p* = 0.4; Table 1).

Maternal age at delivery, yearly household income, maternal education level, and phthalate biomarkers differed among the regions (Seoul, Cheonan, Ulsan), but birth weight, breast-feeding status, gestational age at urine collection, and creatinine levels did not (Table 1).

*Prenatal phthalate levels and MDI and PDI.* First, we performed a sensitivity analysis to assess the impact of excluding subjects with dilute urine samples (< 20 mg/dL creatinine, *n* = 43). Excluding the dilute urine samples had little impact on point estimates but improved the precision of the analysis (data not shown); consequently, the dilute urine samples were excluded from subsequent analyses. Based on models adjusted for key covariates, MDI was inversely associated with a natural log increase in MEHHP (β = –0.97; 95% CI, –1.85 to –0.08), MEOHP (β = –0.95; 95% CI, –1.87 to –0.03), and HHP + OHP (β = –0.98; 95% CI, –1.90 to –0.06); PDI was inversely associated with the MEHHP (β = –1.20; 95% CI, –2.33 to –0.08) (Table 2). Further adjustment for maternal intelligence (based on 227 observations with available data) resulted in significant inverse associations of MDI with MEHHP (β = –1.45; 95% CI, –2.72 to –0.17) and MEOHP (β = –1.54; 95% CI, –2.85 to –0.23) and of PDI increase with MEHHP (β = –1.88; 95% CI, –3.40 to –0.36), MEOHP (β = –1.88; 95% CI, –3.44 to –0.32), and MBP (β = –1.07; 95% CI, –2.10 to –0.03) (Table 2).

**Table 2:** Association of creatinine-corrected prenatal urinary concentrations of phthalate biomarkers on the infant MDI and the psychomotor index of the BSID-II at 6 months.

All cases (*n* = 417)									Subgroup with maternal intelligence (*n* = 227)								
Model 1				Model 2				Model 1				Model 2			
Biomarkers*a*	β*b *(95% CI)		*p*-Value	β*b *(95% CI)		*p*-Value	β*b *(95% CI)		*p*-Value	β*b *(95% CI)		*p*-Value
MDI																				
MEHHP		–1.14	(–2.01 to –0.26)		0.01		–0.97	(–1.85 to –0.08)		0.03		–1.48	(–2.73 to –0.22)		0.02		–1.45	(–2.72 to –0.17)		0.03
MEOHP		–1.13	(–2.04 to –0.23)		0.01		–0.95	(–1.87 to –0.03)		0.04		–1.56	(–2.85 to –0.27)		0.02		–1.54	(–2.85 to –0.23)		0.02
HHP + OHP		–1.12	(–2.03 to –0.21)		0.02		–0.98	(–1.90 to –0.06)		0.04		–1.47	(–2.76 to –0.19)		0.03		–1.15	(–2.45 to 0.15)		0.08
MBP		–0.58	(–1.22 to 0.07)		0.08		–0.54	(–1.18 to 0.10)		0.10		–0.73	(–1.58 to 1.13)		0.10		–0.64	(–1.51 to 0.23)		0.15
PDI																				
MEHHP		–1.31	(–2.45 to –0.17)		0.03		–1.20	(–2.33 to –0.08)		0.04		–2.00	(–3.52 to –0.47)		0.01		–1.88	(–3.40 to –0.36)		0.02
MEOHP		–1.01	(–2.19 to 0.17)		0.09		–0.92	(–2.10 to 0.26)		0.13		–2.03	(–3.60 to –0.46)		0.01		–1.88	(–3.44 to –0.32)		0.02
HHP + OHP		–1.23	(–2.41 to –0.05)		0.04		–1.48	(–3.04 to 0.07)		0.06		–2.03	(–3.57 to –0.49)		0.01		–1.51	(–3.06 to 0.04)		0.06
MBP		–0.77	(–1.61 to 0.06)		0.07		–0.79	(–1.60 to 0.03)		0.06		–1.06	(–2.10 to 2.10)		0.05		–1.07	(–2.10 to –0.03)		0.04
Model 1: unadjusted. Model 2: adjusted for infant birth weight, infant sex, maternal age, maternal education level, family income, breast-feeding status, and residential area (plus maternal intelligence for subgroup analysis). **a**Prenatal maternal urinary concentrations were natural log-transformed. Urine samples with creatinine values > 20 mg/dL were used. **b**MDI or PDI scores per natural log change in micrograms per gram creatinine.																				

Because phthalates may affect males and females differently, we performed analyses for male and female infants separately. In male infants, MDI was inversely associated with a natural log increase in MEHHP (β = –1.46; 95% CI, –2.70 to –0.22), MEOHP (β = –1.57; 95% CI, –2.87 to –0.28), HHP + OHP (β = –1.57; 95% CI, –2.86 to –0.28), and MBP (β = –0.93; 95% CI, –1.82 to –0.05); PDI was inversely associated with MEHHP (β = –2.36; 95% CI, –3.94 to –0.79), MEOHP (β = –2.05; 95% CI, –3.71 to –0.39), HHP + OHP (β = –2.28; 95% CI, –3.93 to –0.63), and MBP (β = –1.25; 95% CI, –2.40 to –0.11) (Table 3). Inverse associations between phthalate biomarkers and developmental indices at 6 months were also observed in females, but the association was weaker and did not reach statistical significance (Table 3). The differences between males and females were significant (*p* < 0.10) for PDI in associations with MEHHP and HHP + OHP only (Table 3).

**Table 3:** Association of creatinine-corrected prenatal urinary concentrations of phthalate biomarkers on the infant MDI and PDI of the BSID-II at 6 months according to sex.

Male (*n* = 211)									Female (*n* = 206)								
Model 1				Model 2				Model 1				Model 2				*p*-Value for interaction*c*
Biomarkers*a*	β*b *(95% CI)		*p*-Value	β*b *(95% CI)		*p-*Value	β*b *(95% CI)		*p*-Value	β*b *(95% CI)		*p-*Value				
MDI																						
MEHHP		–1.41	(–2.63 to –0.18)		0.03		–1.46	(–2.70 to –0.22)		0.02		–088	(–2.15 to 0.38)		0.17		–0.56	(–1.87 to 0.75)		0.40		0.27
MEOHP		–1.55	(–2.82 to –0.29)		0.02		–1.57	(–2.87 to –0.28)		0.02		–0.73	(–2.04 to 0.57)		0.27		–0.43	(–1.79 to 0.94)		0.54		0.18
HHP+OHP		–1.58	(–2.86 to –0.31)		0.02		–1.57	(–2.86 to –0.28)		0.02		–0.69	(–2.00 to 0.62)		0.30		–0.49	(–1.85 to 0.86)		0.47		0.21
MBP		–0.79	(–1.68 to 0.10)		0.08		–0.93	(–1.82 to –1.82)		0.04		–0.37	(–1.31 to 0.57)		0.44		–0.21	(–1.17 to 0.75)		0.66		0.30
PDI																						
MEHHP		–2.00	(–3.58 to –0.43)		0.01		–2.36	(–3.94 to –0.79)		< 0.01		–0.60	(–2.24 to 1.04)		0.47		–0.29	(–1.94 to 1.37)		0.73		0.07
MEOHP		–1.63	(–3.27 to 0.01)		0.05		–2.05	(–3.71 to –0.39)		0.02		–0.37	(–2.06 to 1.32)		0.67		–0.08	(–1.79 to 1.64)		0.93		0.11
HHP+OHP		–1.90	(–3.53 to –0.27)		0.02		–2.28	(–3.93 to –0.63)		0.01		–0.55	(–2.24 to 1.15)		0.52		–0.16	(–1.87 to 1.55)		0.85		0.08
MBP		–1.02	(–2.17 to 0.13)		0.08		–1.25	(–2.40 to –0.11)		0.03		–0.51	(–1.73 to 0.71)		0.41		–0.42	(–1.63 to 0.78)		0.49		0.30
Model 1: unadjusted. Model 2: adjusted for infant birth weight, maternal age, maternal education level, family income, breast-feeding, and residential area. **a**Prenatal maternal urinary concentrations were natural log-transformed. Urine samples with creatinine values > 20 mg/dL were used. **b**MDI or PDI scores per natural log change in micrograms per gram creatinine. **c**Adjusted for infant birth weight, maternal age, maternal education level, family income, breast-feeding status, residential area, infant sex, and the infant sex–phthalate interaction.																						

**Figure 1:**
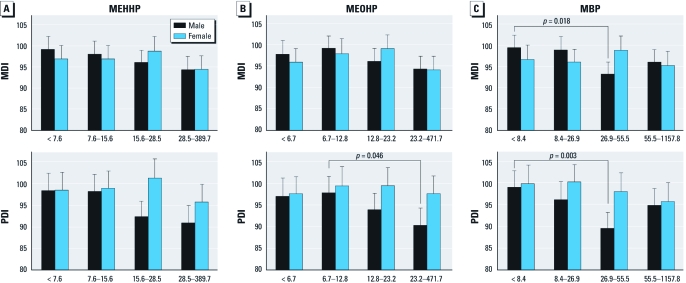
Adjusted mean MDI and PDI scores according to quartiles of creatinine-corrected prenatal phthalate metabolite concentrations in maternal urine (based on samples with urine creatinine > 20 mg/dL) adjusted for birth weight, maternal age, maternal education level, family income, breast-feeding status, and residential area. Error bars represent the 95% CIs of the predicted mean. The *p*-value for the infant sex–phthalate interaction was significant only for MBP and the MDI (*p* = 0.03). Among the male infants, there were significant group differences in the MDI scores among the MBP quartiles (*p* = 0.01) and in the PDI scores among the MEHHP (*p* = 0.06), MEOHP (*p* = 0.04), and MBP quartiles (*p* = 0.01). Among the female infants, there were no significant group differences among the phthalate quartiles for either the MDI or the PDI scores. Values for the first, second, third, and fourth quartiles, respectively, were as follows: MEHHP: < 7.6, 7.6 to 15.6, 15.6 to 28.5, and 28.5 to 389.7 µg/gC (micrograms per gram creatinine); MEOHP: < 6.7, 6.7 to 12.8, 12.8 to 23.2, and 23.2 to 471.7 µg/gC; MBP: < 8.4, 8.4 to 26.9, 26.9 to 55.5, and 55.5 to 1157.8 µg/gC.

*Quartiles of creatinine-corrected urine concentrations of phthalate biomarkers and MDI and PDI.* We conducted a categorical analysis to estimate associations of MDI and PDI with quartiles of creatinine-corrected phthalate biomarkers according to infant sex (Figure 1). The model was adjusted for infant birth weight, maternal age, maternal education level, family income, breast-feeding status, and residential area. Among female infants, there were no significant differences in MDI or PDI scores among MEHHP quartiles (Figure 1A). Differences between males and females were not statistically significant, but among male infants, MDI and PDI scores decreased with increasing MEHHP quartiles, with a significant overall association for PDI (*p* = 0.01). Male infants in the fourth MEHHP quartile showed lower PDI scores than did those in the first quartile (*p* = 0.06).

Among female infants, MDI and PDI scores were not significantly different among MEOHP quartiles (Figure 1B). Differences between males and females were not statistically significant, but among male infants the MDI and PDI scores decreased with the increase in MEOHP quartiles, with significant differences among quartiles for PDI (*p* = 0.04).

There were significant group differences in MDI (*p* = 0.01) and PDI (*p* < 0.01) scores among MBP quartiles in males (Figure 1C), with the lowest scores among those in the third quartile for both outcomes. Among the female infants, there were no significant group differences in MDI or PDI scores among MBP quartiles, although PDI scores tended to decrease. In addition, overall associations between MBP quartiles and the MDI score differed significantly between males and females (*p* = 0.03).

## Discussion

We found an inverse association between prenatal exposure to phthalates and the MDI and PDI scores of infants at 6 months. In this study, we observed a strong inverse association for male infants between all measured phthalate metabolites (MEHHP, MEOHP, and MBP) and both developmental indices, whereas none of the associations were significant in female infants. We also observed significant differences between males and females in the association between PDI and natural log-transformed MEHHP and the association between MDI and MBP quartiles. These results suggest that infant sex modified associations between phthalates and developmental scores at 6 months. Sex-specific effects on orientation and motor domains on the Brazelton Neonatal Behavioral Assessment Scale have been reported in neonates, with male and female infants showing mirror image patterns of each other (Engel et al. 2009). Prenatal exposure to phthalates has been associated with reduced masculine play among 3- to 6-year-old boys, as measured by the Pre-School Activities Inventory (Swan et al. 2010). However, no sex-specific differences were observed in the association between prenatal phthalate exposure and childhood social impairment at 7–9 years (Miodovnik et al. 2011). Therefore, follow-up studies are warranted to determine if differences in neurodevelopmental effects of phthalates between males and females persist in older children.

At 6 months, the BSID-II assesses visual and auditory attention, visual memory, recognition and imitation of simple sounds, and gross and fine motor skills (Bayley 1993). These developmental domains are different from intelligence or executive function, which can be measured only at a later age, making it difficult to compare this study with studies conducted in older children. However, Engel et al. (2010) reported that prenatal exposure to phthalates was associated with externalizing behavior problems (β = 1.24–2.40) on the Behavioral Assessment System for Children-Parent Rating Scales and poor executive function (β = 1.23) on the Behavioral Rating Inventory of Executive Function at 4–9 years of age. Prenatal exposure to phthalates has also been associated with social impairment on the Social Responsiveness Scale (β = 1.40–1.86) at 7–9 years of age (Miodovnik et al. 2011). A recent cross-sectional study also reported an inverse association between vocabulary subscores on intelligence tests and concurrent urine phthalate levels (β = 0.44–0.53) at 8–11 years of age (Cho et al. 2010). The estimated effect sizes reported for previous studies were slightly lower than or similar to those for PDI (β = 1.20) and MDI (β = 0.95–0.98) in 6-month-old infants in our cohort.

Environmental exposure to phthalates in humans may contribute to adverse neurodevelopmental outcomes in several ways. Phthalates may interfere with the thyroid hormone system (Ghisari and Bonefeld-Jorgensen 2009; Huang et al. 2007) or the lipid signal transduction pathways that may influence the development of cognitive function (Xu et al. 2007). Phthalates have been shown to cause hyperactivity in rats, possibly through effects on the dopamine system (Ishido et al. 2004a, 2004b). Phthalates have been shown to decrease the number of midbrain dopaminergic neurons, tyrosine hydroxylase biosynthetic activity (Tanida et al. 2009), and tyrosine hydroxylase immunoreactivity (Ishido et al. 2004b). Phthalates also exhibit antiandrogenic activity (Borch et al. 2006), which may interfere with the regulation of normal fetal brain development (Colborn 2004). However, there is still limited knowledge about the etiological mechanism underlying the possible detrimental effects of phthalates on the development of human brain.

The phthalate exposures in this study were generally of the same magnitude as those reported by other studies of pregnant women. Median MEHHP (10.1 µg/L), MEOHP (7.9 µg/L), and MBP (16.6 µg/L) concentrations in this study were somewhat lower than the median levels in pregnant women in the Children’s Environmental Health study (20.0, 17.0, and 36.0 µg/L, respectively) (Wolff et al. 2008). Geometric mean concentrations of MEHHP (8.9 µg/L), MEOHP (7.4 µg/L), and MBP (12.4 µg/L) in this study were slightly lower than those in pregnant women in the NHANES 2001–2002 study (19.2, 15.6, and 19.8 µg/L, respectively) (Ye et al. 2009). Ethnic and social differences and differences in the data collection time points must be taken into account when making comparisons among study populations. Median concentrations of MEHHP, MEOHP, and MBP reported for Korean women > 20 years of age (13.2, 11.2, and 44.8 µg/L, respectively) (Lee et al. 2008) and for pregnant Japanese women (10.6, 11.0, and 57.9 µg/L, respectively) (Suzuki et al. 2009) were higher than median concentrations in our population of pregnant Korean women.

The limitations of this study need to be considered. First, the possibility that mothers with large muscle mass and consequently high urinary creatinine concentrations may bear larger babies with higher scores in the development index, resulting in the inverse associations seen in this study, must be considered. However, such systemic bias would have resulted in an inverse relationship in both male and female babies. Furthermore, no association was observed between the creatinine concentration and demographic characteristics, including the birth weight of the child (data not shown). We have also adjusted for several potential confounders, including maternal intelligence, a well-known predictor of neurodevelopment (Mink et al. 2004). Adjusting for these factors generally strengthened associations between exposure to phthalates and developmental scores.

Although the spot urine sample in this study was collected between 0900 and 1800 hours, the exact time of the day was unavailable, making it difficult to assess the effect of time of urine collection on phthalate concentration. There have been concerns raised over whether single spot urine tests truly represent the long-term prenatal exposure to phthalates because of the short half-lives of phthalates and the episodic nature of the exposure. However, a recent study showed that the phthalates detected in the spot urine of pregnant women at 25–40 weeks of gestation reasonably reflected exposure for approximately 2 months (Suzuki et al. 2009).

Finally, we measured only three phthalate metabolites: two metabolites of DEHP (MEHHP and MEOHP) and one metabolite of DBP (MBP). Previous studies have shown that the metabolites of DEHP and DBP are associated with thyroid dysfunction in pregnant women (Huang et al. 2007), but these compounds continue to be widely used in wall coverings, car interiors, clothing, and toys (Bornehag et al. 2005). The three metabolites were chosen after careful consideration of exposure amounts, sample availability, and the results of previous studies (Cho et al. 2010; Engel et al. 2009; Kim BN et al. 2009). However, additional studies using multiple prospective measurements and larger sample sizes are needed to expand the interpretation of our results to other phthalates.

In this study, we hypothesized that prenatal exposure to DEHP and DBP would be inversely associated with MDI and PDI as measured by the BSID-II. This study observed a strong inverse association between prenatal exposure to MEHHP, MEOHP, and MBP in the third trimester of pregnancy and the MDI and PDI of the male infants at 6 months. These findings add further support to the possibility that prenatal phthalate exposure may be detrimental to neurodevelopment and suggest possible sex differences in the sensitivity to phthalates.
